# Supplementary material for: Antibody neutralization of SARS-CoV-2 through ACE2 receptor mimicry
Source: Nat Commun. 2021 Jan 11;12:250. doi: 10.1038/s41467-020-20501-9 (PMC7801515; doi:10.1038/s41467-020-20501-9)
Supplement: Supplementary file 1 — Supplementary Information [file 41467_2020_20501_MOESM1_ESM.pdf]

Antibody neutralization of SARS-CoV-2 through ACE2 receptor mimicry

Jiwan Ge[1,*], Ruoke Wang[2,*], Bin Ju[3,*], Qi Zhang[2,*], Jing Sun[4], Peng Chen[2], Senyan Zhang[1], Yuling Tian[1], Sisi Shan[2], Ling Cheng[3], Bing Zhou[3], Shuo Song[3], Juanjuan Zhao[3], Haiyan Wang[3], Xuanling Shi[2], Qiang Ding[2], Lei Liu[3], Jincun Zhao[4], Zheng Zhang[3,#], Xinquan Wang[1,#] and Linqi Zhang[2,#]

[1]The Ministry of Education Key Laboratory of Protein Science, Beijing Advanced Innovation Center for Structural Biology, Beijing Frontier Research Center for Biological Structure, Collaborative Innovation Center for Biotherapy, School of Life Sciences, Tsinghua University, Beijing 100084, China

[2]Comprehensive AIDS Research Center, Beijing Advanced Innovation Center for Structural Biology, School of Medicine and Vanke School of Public Health, Tsinghua University, Beijing 100084, China

[3]Institute for Hepatology, National Clinical Research Center for Infectious Disease, Shenzhen Third People's Hospital; The Second Affiliated Hospital, School of Medicine, Southern University of Science and Technology, Shenzhen 518112, Guangdong Province, China

[4]State Key Laboratory of Respiratory Disease, National Clinical Research Center for Respiratory Disease, Guangzhou Institute of Respiratory Health, the First Affiliated Hospital of Guangzhou Medical University, Guangzhou, Guangdong 510182, China

*These authors contributed equally to this work.

#Correspondence:

zhangzheng1975@aliyun.com (Z.Z)

xinquanwang@mail.tsinghua.edu.cn (X.W.)

zhanglinqi@tsinghua.edu.cn (L.Z.)

Supplementary information
Supplementary Figures 1-8
Supplementary Tables 1-3

a

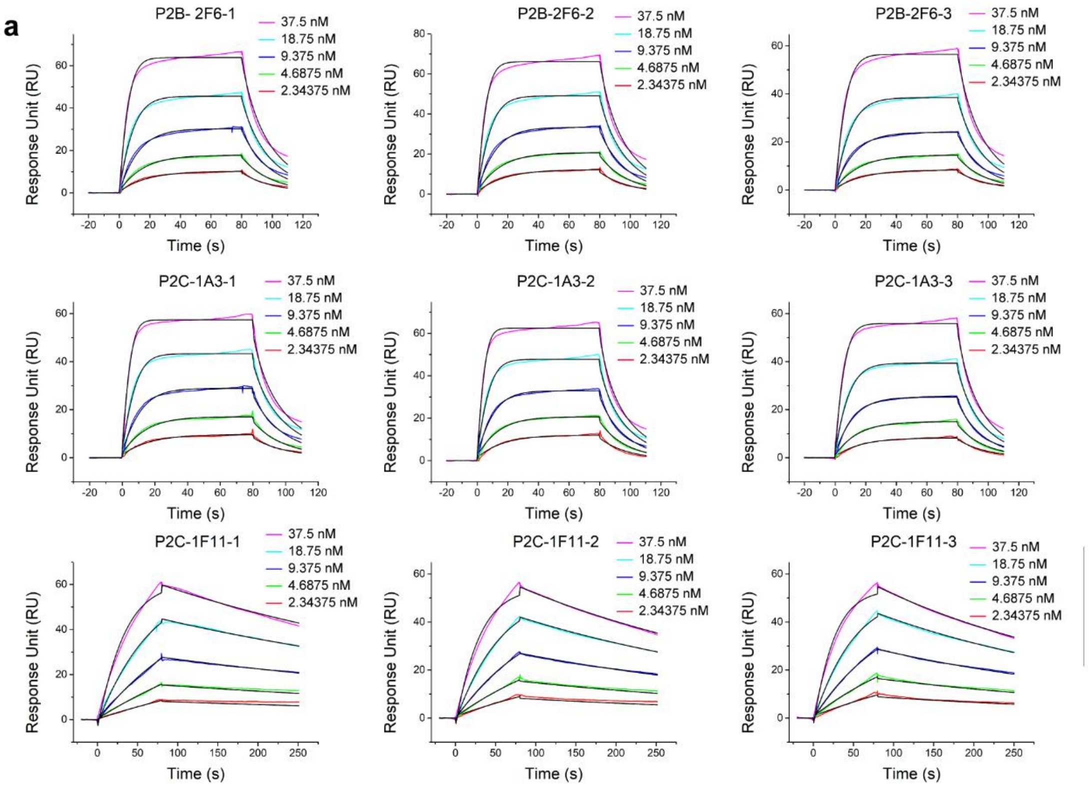

b

	ka (1/Ms)	kd (1/s)	K_D (nM)	K_D (nM)
**P2B-2F6-1**	6.478E+6	0.09106	14.06	15.62 ± 2.94
**P2B-2F6-2**	7.407E+6	0.1022	13.80	
**P2B-2F6-3**	5.383E+6	0.1023	19.01	
**P2C-1A3-1**	1.115E+7	0.1029	9.23	12.24 ± 4.05
**P2C-1A3-2**	1.181E+7	0.1258	10.65	
**P2C-1A3-3**	6.985E+6	0.1177	16.85	
**P2C-1F11-1**	1.944E+6	0.002129	1.10	1.72 ± 0.84
**P2C-1F11-2**	1.905E+6	0.002625	1.38	
**P2C-1F11-3**	1.133E+6	0.003031	2.67	

Supplementary Fig. 1 Measurement of K_D by SPR. (**a**) Binding curves of immobilized neutralizing antibody (P2B-2F6, P2C-1A3 and P2C-1F11) to the SARS-CoV-2 RBD. Data are presented by colored lines and the best fit of the data to a 1:1 binding model are shown in black. (**b**) Summary of the binding kinetics between SARS-CoV-2 RBD and the neutralizing antibodies measured in three independent experiments.

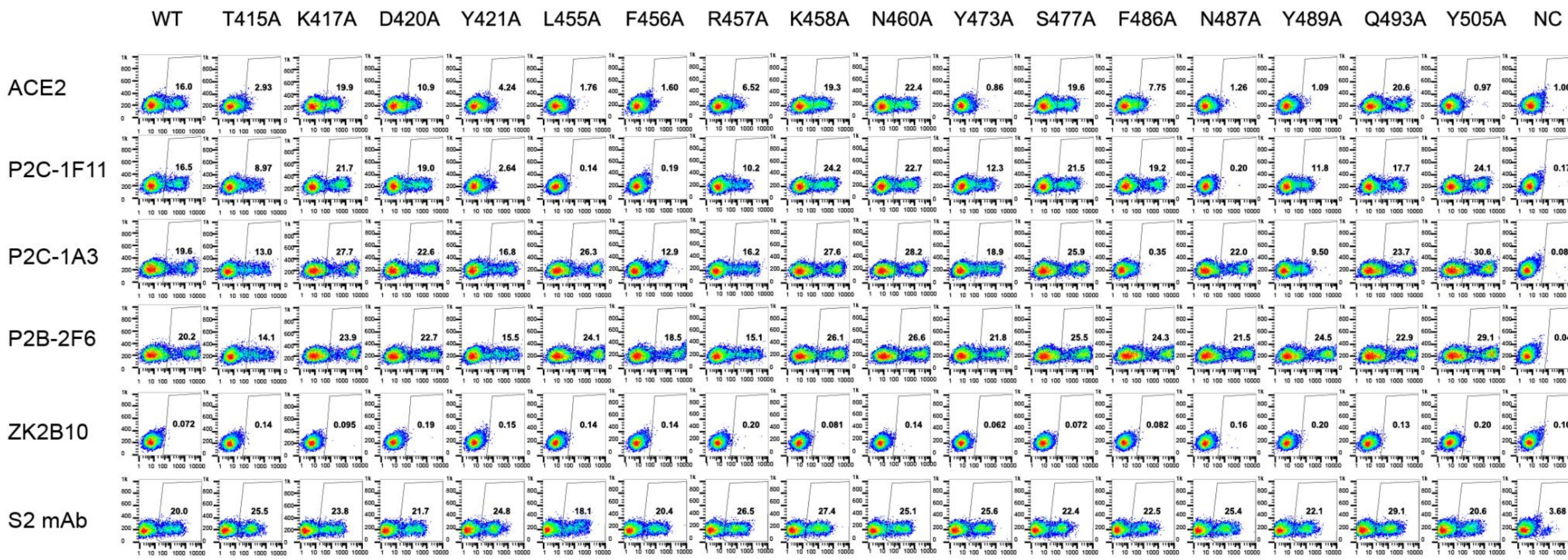
WT
T415A
K417A
D420A
Y421A
L455A
F456A
R457A
K458A
N460A
Y473A
S477A
F486A
N487A
Y489A
Q493A
Y505A
NC
ACE2
16.0 2.93 19.9 10.9 4.24 1.76 1.60 6.52 19.3 22.4 0.86 19.6 7.75 1.26 1.09 20.6 0.97 1.00
P2C-1F11
16.5 8.97 21.7 19.0 2.64 0.14 0.19 10.2 24.2 22.7 12.3 21.5 19.2 0.20 11.8 17.7 24.1 0.17
P2C-1A3
19.6 13.0 27.7 22.6 16.8 26.3 12.9 16.2 27.6 28.2 18.9 25.9 0.35 22.0 9.50 23.7 30.6 0.082
P2B-2F6
20.2 14.1 23.9 22.7 15.5 24.1 18.5 15.1 26.1 26.6 21.8 25.5 24.3 21.5 24.5 22.9 29.1 0.041
ZK2B10
0.072 0.14 0.095 0.19 0.15 0.14 0.14 0.20 0.081 0.14 0.062 0.072 0.082 0.16 0.20 0.13 0.20 0.10
S2 mAb
20.0 25.5 23.8 21.7 24.8 18.1 20.4 26.5 27.4 25.1 25.6 22.4 22.5 25.4 22.1 29.1 20.6 3.68

Supplementary Fig. 2 Binding of recombinant ACE2 and the nAbs to HEK 293T cell surface expressed wild-type or 16 single alanine mutated S of SARS-CoV-2. For each panel, X-axis means ACE2 or tested antibody binding-PE/APC and Y-axis means SSC. The percentage of positive cells are highlighted in the gate. S2 is a positive control antibody used for S expression normalization while ZK2B10 is negative control antibody specific for DIII of Zika virus glycoprotein. Mock transfected cells (NC) were used as the background control. The panel shows one representative experiment from data shown in Fig. 3**a-d**.

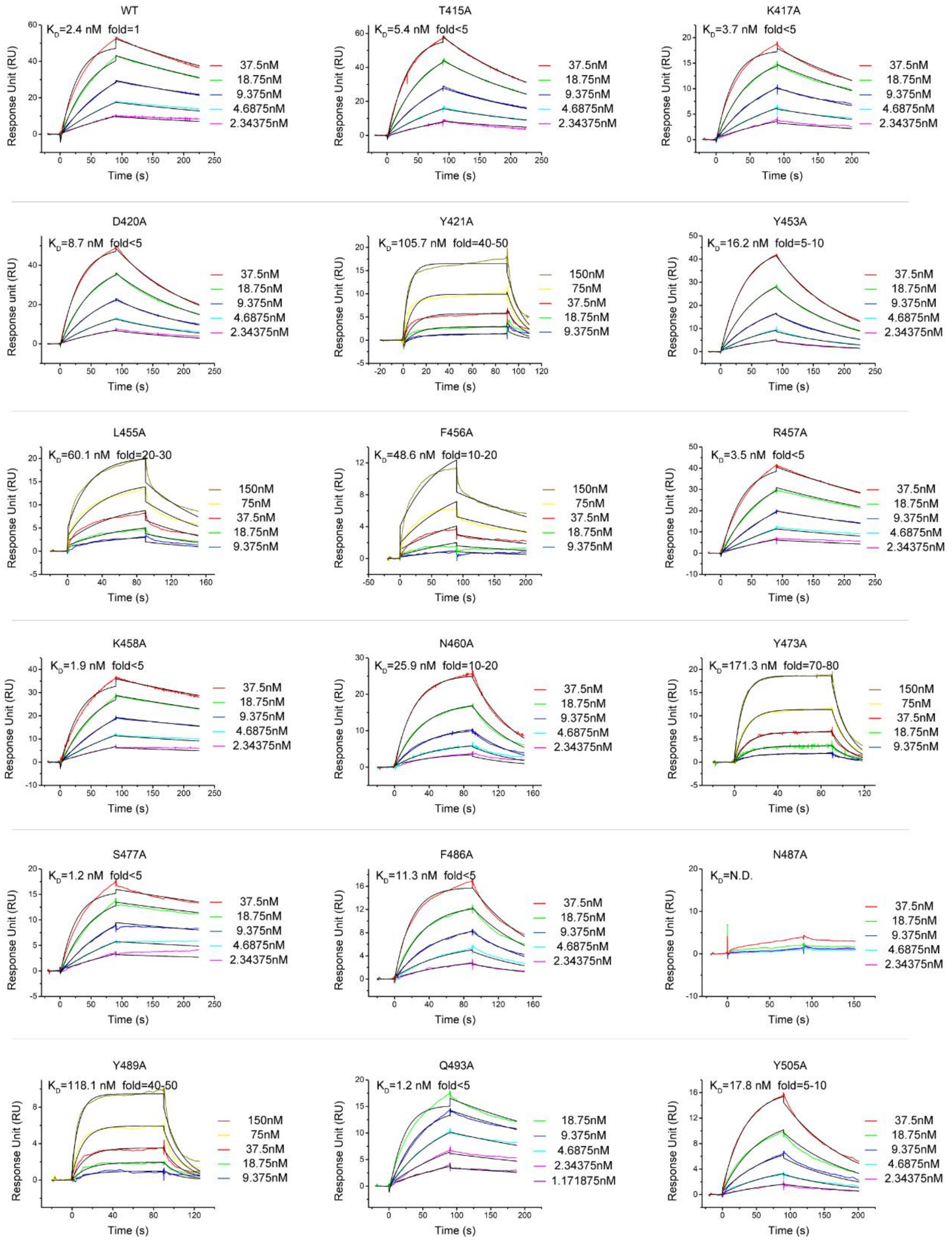

Supplementary Fig. 3 Binding kinetics of P2C-1F11 to wild-type or mutant SARS-CoV-2 RBD measured by SPR. P2C-1F11 was immobilized on the CM5 sensor and wild type or mutant SARS-CoV-2 RBD was flowed through the system in serial concentrations. Colored lines indicate the experimentally derived curves. Black lines represent best fitted curves based on the experimental data. The calculated KD and fold changes for P2C-1F11 binding to each mutant RBD relative to those of wild-type are indicated.

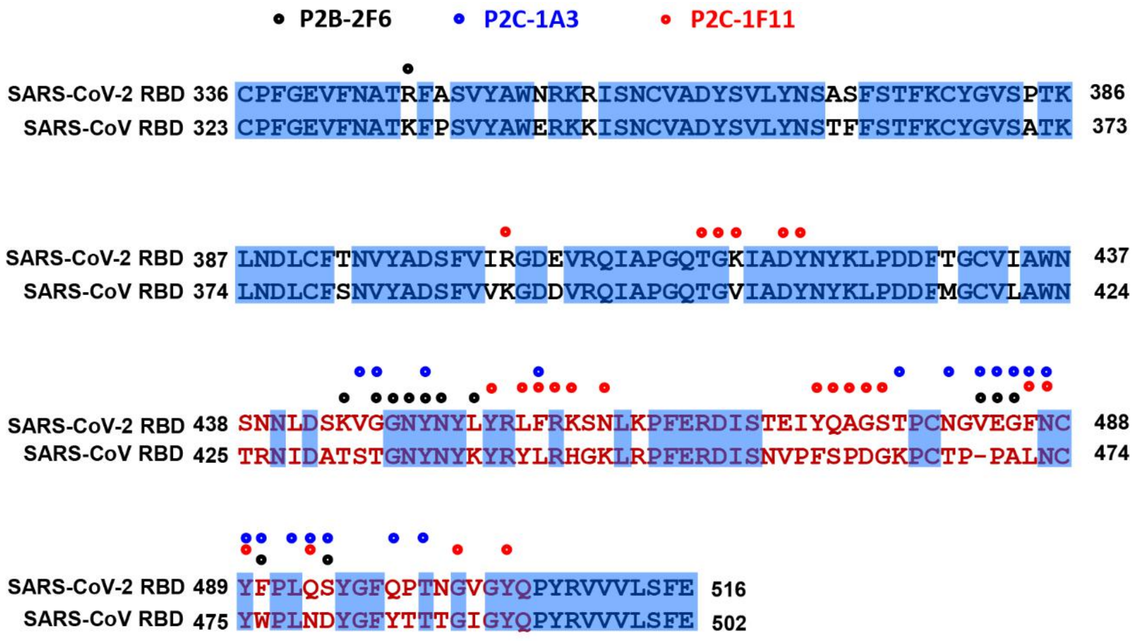

Supplementary Fig. 4 Comparing epitope residues of P2C-1F11, P2C-1A3 and P2B-2F6 along the SARS-CoV-2 and SARS-CoV RBD alignments. Epitope residues of P2C-1F11, P2C-1A3 and P2B-2F6 are indicated by red, blue and black circles, respectively. Blue boxes highlight conserved residues between the two RBD sequences. Red residues are those located within the receptor binding motif (RBM).

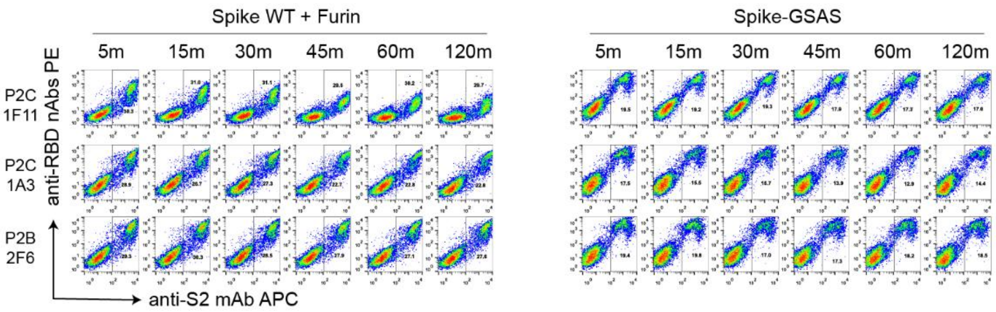

Supplementary Fig. 5 Dynamics of S1, S2, S1/S2 ratios on the cell surface measured by flow cytometry at 37°C with co-transfection of the plasmid encoding Furin protease, (left) or with a mutated S containing GSAS substitution at S1/S2 cleavage motif (right). An anti-S2 antibody were added at the end of incubation with the testing antibodies and detected simultaneously. The panel shows one representative experiment from data shown in Fig. 4**a**-**f**.

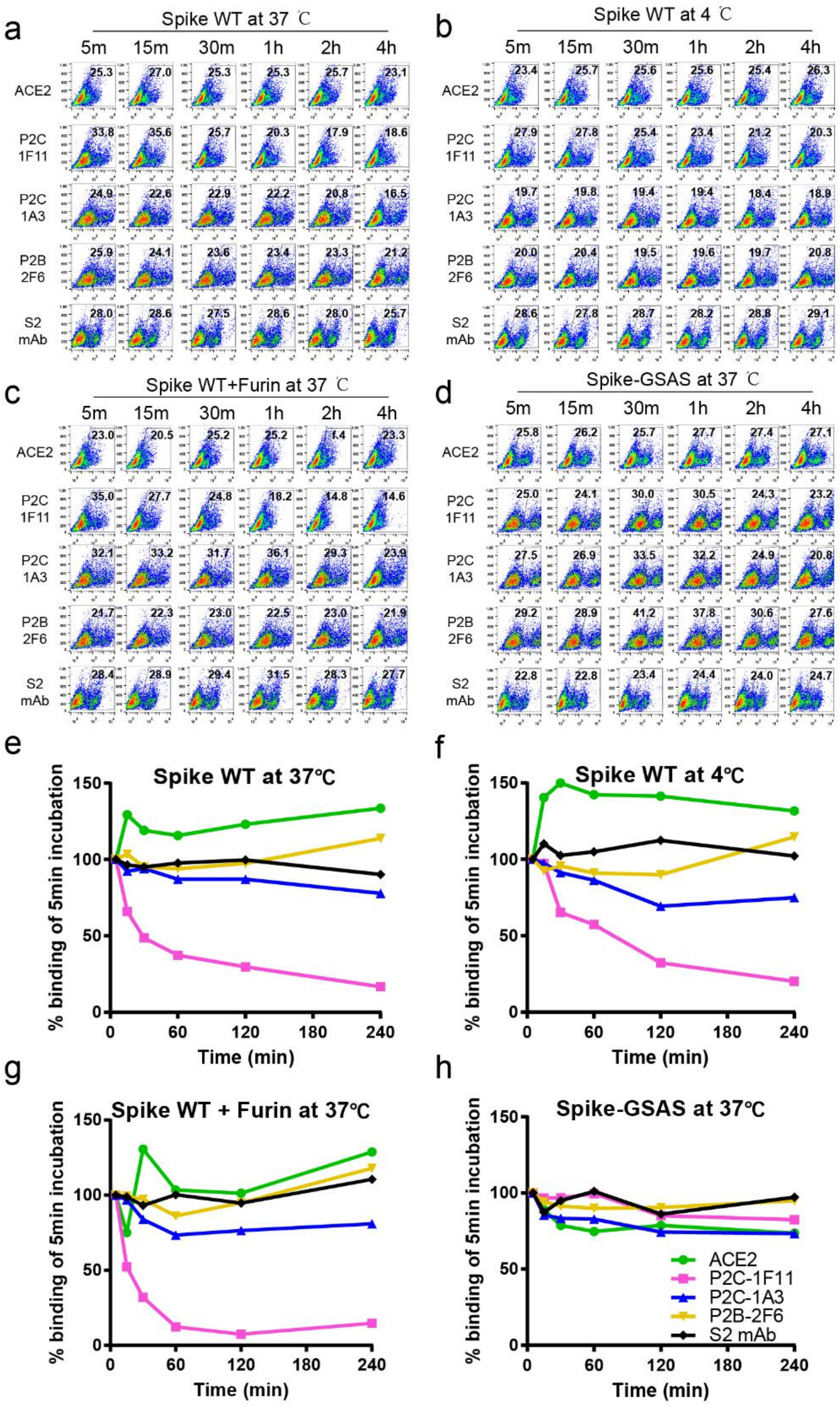

Supplementary Fig. 6 Shedding of S1 over time measured by flow cytometry (**a, e**) at 37°C, (**b, f**) at 4°C, (**c, g**) at 37°C with co-transfection of the plasmid encoding Furin protease, or (**d, h**) at 37°C with a mutated S containing GSAS substitution at S1/S2 cleavage motif. For each panel of fluorescence, X-axis means ACE2 or tested antibody binding-PE or -FITC and Y-axis means SSC. Data shown were representative of at least two independent experiments.

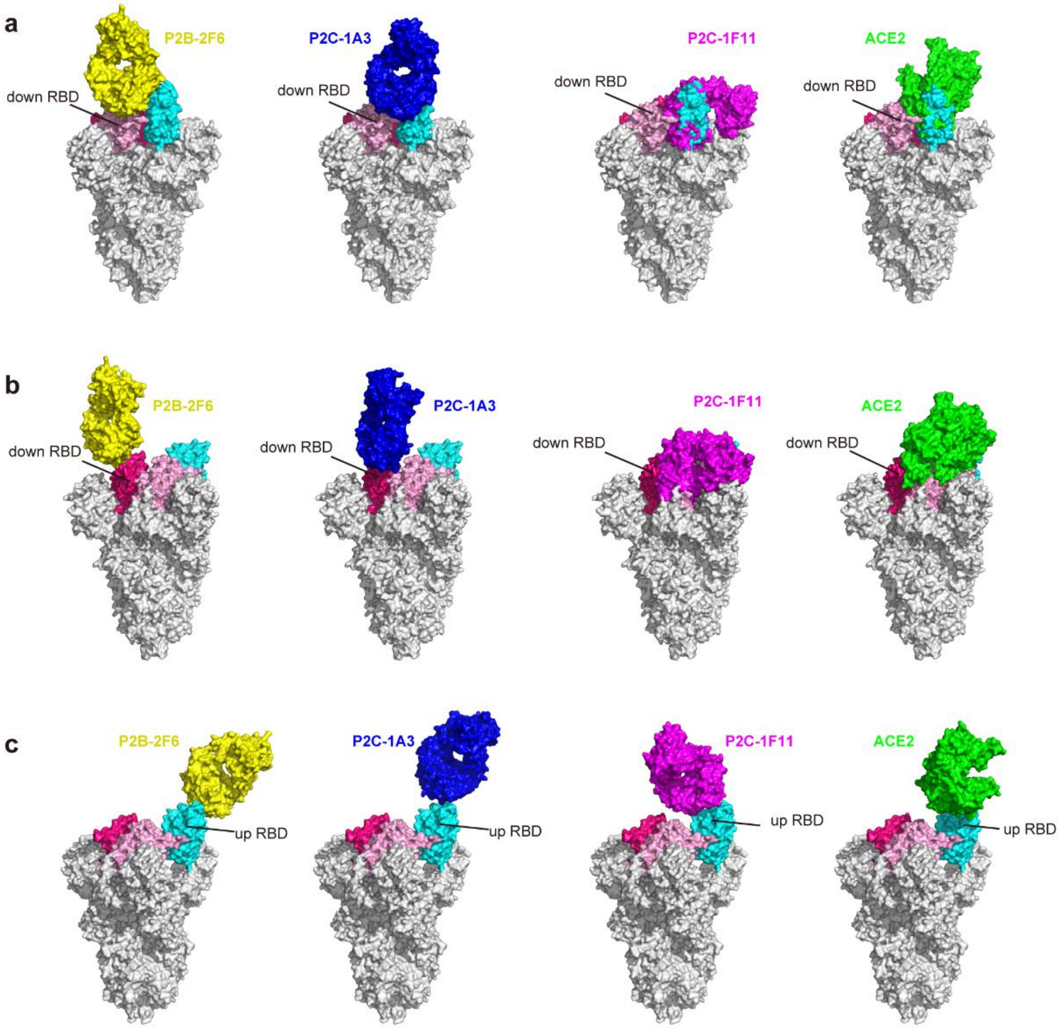

Supplementary Fig. 7 Antibody and ACE2 binding models to different RBD conformations in the context of trimeric Spike. The SARS-CoV-2 S trimer is shown in grey with one RBD in "up" (cyan) and two RBDs in "down" (pink and hot pink) conformations (PDB ID: 6VSB). (**a**,**b**) RBD-P2B-2F6 Fab and RBD-P2C-1A3 Fab can align with the "down" conformation whereas RBD-P2C-1F11 Fab and RBD-ACE2 cannot due to heavy spatial clash with the S trimer. (**c**) All RBD-P2B-2F6 Fab, RBD-P2C-1A3 Fab, RBD-P2C-1F11 Fab, and RBD-ACE2 align to the "up" RBD conformation. The P2B-2F6 Fab, P2C-1A3 Fab, P2C-1F11 Fab and ACE2 are colored in yellow, blue, magenta and green, respectively.

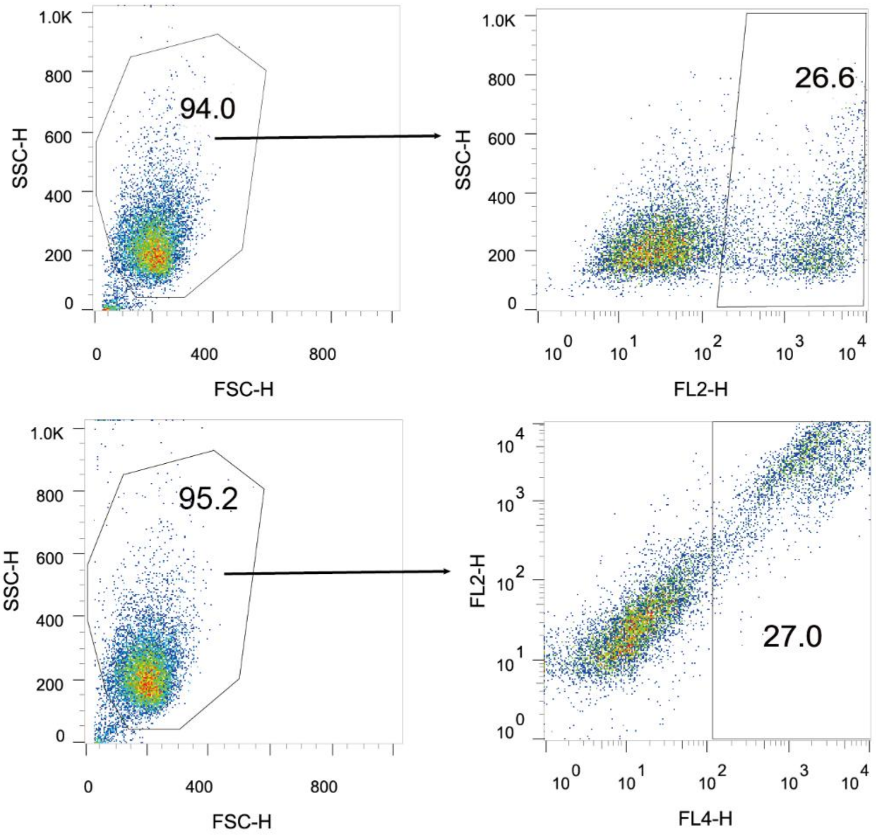

Supplementary Fig. 8 Gating strategy used for cell-surface staining analysis.
Upper gating strategy were used in the experiment to study the impact of single Alanine mutated S on the antibody binding activity (Figure 3**a-d**, Supplementary Fig. 2). HEK 293T cells transfected with plasmids encoding S or mutated S were incubated with ACE2 or antibodies, and stained with anti-his PE, anti-human IgG Fc PE or anti-mouse IgG FITC. Lower Gating strategy were used in the experiment to analyze the shedding process (Figure 4**a-f**, supplementary Fig. 5). HEK 293T cells transfected with plasmids encoding wild typed S or GSAS-S were incubated with tested antibodies, and stained with anti-human IgG Fc PE and anti-mouse IgG Fc APC simultaneously.

Supplementary Table 1. Data collection and refinement statistics.

**Data collection**		
	**SARS-CoV-2 RBD-P2C-1F11 complex**	**SARS-CoV-2 RBD-P2C-1A3 complex**
Wavelength (Å)	0.97918	0.97918
Resolution range (Å)	34.29-2.96 (3.04-2.96) *	50.00-3.40 (3.48-3.40) *
Space group	*C*2	$P6_122$
Unit cell dimensions		
a, *b*, *c* (Å)	194.88, 85.39, 58.51	89.411, 89.411, 437.923
α, β, γ (°)	90, 100.29, 90	90, 90, 120
Unique reflections	19785 (1951)	15108 (1332)
Completeness (%)	99.68 (99.90)	99.74 (89.69)
Mean I/sigma (I)	10.1 (1.8)	8.8 (1.2)
Redundancy	6.8 (6.9)	8.7 (7.9)
R_{merge} (%)	15.5 (99.7)	11.8 (93.3)
R_{pim} (%)	9.7 (62.4)	8.4 (45.7)
CC1/2	0.994 (0.752)	0.990 (0.796)
Wilson B-factor (Å^2)	61.70	105.13

**Structure refinement**		
Resolution (Å)	29.03-2.96	20.14-3.40
R_{work} /R_{free} (%)	20.2/25.3	23.5/28.3
No. atoms		
Protein	4697	4763
Ligands	14	14
Protein residues	616	621
B-factors (Å^2)		
Protein	60.95	125.59
Ligands	99.03	132.45
RMSD		
Bonds length (Å)	0.008	0.009
Bonds angles (°)	1.02	1.09
Ramachandran plot		
Favored (%)	95.21	91.99
Allowed (%)	4.63	8.01
Outliers (%)	0.17	0.00

*One crystal was used for the data.

*Values in the parentheses are for high-resolution shell.

Supplementary Table 2. Contacts between SARS-CoV-2 RBD and P2C-1F11 or P2C-1A3 (distance cutoff 4Å).

P2C-1F11				P2C-1A3				P2B-2F6			
RBD	Heavy chain	RBD	Light chain	RBD	Heavy chain	RBD	Light chain	RBD	Heavy chain	RBD	Light chain
T415	S56, Y58	R403	Y33	V445	D73	T478	S28, Y30, N90	R346	H54	V483	G31, Y32. N33
G416	Y52, Y58	Y453	Y33	G446	R72, D73	N481	Q25, S91	K444	S31	E484	N33, Y34
K417	Y52	G502	S28	Y449	S54, R72, D73, N74	V483	S91	G446	Y27	G485	N33
D420	Y52, S56	Y505	S28, V29, S30, Q91, Y92	F456	H102	E484	S91, Y92	G447	Y27, S31		
Y421	Y33, Y52, S53, G54			E484	Y33, Y50, T57	G485	N90	N448	S31		
L455	Y33, V101			G485	F100	F486	Y30, L89	Y449	S31, G102, I103, Y27, Y33		
F456	Y33, L99			F486	F100, S101, L105	N487	Y30	N450	H54, S30, S31		
R457	S53			Y489	Y33, F100, S101, H102			L452	V105, I103		
K458	S31, S53, G54			F490	T57			E484	R112		
N460	G54			L492	S56			F490	P107, V105		
Y473	S31, S53			Q493	S53, S54, H102			S494	I103		
Q474	S31			S494	S54, S56						
A475	I27, T28, N32, R97			Q498	D73, N74, A75						
G476	T28			T500	A75						
S477	T28										
F486	R97, D105										
N487	G26, I27, R97										
Y489	R97, L99										
Q493	Y102										

Supplementary Table 3. Primers used in this manuscript.

Name	Sequence (5'-3')
RBD-319F-BamHI-B12	CTGCCTTTGCGGCGGATCCCAGAGTGCAGCCTACCGAG
RBD-529R-HindIII-B12	AGTACTTCTCGACAAGCTTCTAATGGTGATGGTGATGGTGCTTCTTAGGGCCGCACAC
spike-F	GGTACCGAGCTCGGATCCGGATCCACCACCATGTTCGTGTTCCTGGTG
spike-R	CTGTGCTGGATATCTGCAGAATTCTCATTAGGTGTAGTGCAGCTTCACG
T415A-F	GGCAGGCGGGCAAGATCGCCGACTAC
T415A-R	TTGCCCGCCTGCCCTGGCGCGATCTG
D420A-F	TCGCCGCGTACAATTACAAGCTGCCT
D420A-R	TTGTACGCGGCGATCTTGCCGGTCTG
Y421A-F	CCGACGCGAATTACAAGCTGCCTGAC
Y421A-R	TAATTCGCGTCGGCGATCTTGCCGGT
Y453A-F	ACCTGGCGAGACTGTTCAGAAAGAGC
Y453A-R	AGTCTCGCCAGGTAATTGTAATTGCC
L455A-F	ACAGAGCGTTCAGAAAGAGCAATCTG
L455A-R	CTGAACGCTCTGTACAGGTAATTGTA
F456A-F	GACTGGCGAGAAAGAGCAATCTGAAG
F456A-R	TTTCTCGCCAGTCTGTACAGGTAATT
R457A-F	TGTTCGCGAAGAGCAATCTGAAGCCT
R457A-R	CTCTTCGCGAACAGTCTGTACAGGTA
K458A-F	TCAGAGCGAGCAATCTGAAGCCTTTC
K458A-R	TTGCTCGCTCTGAACAGTCTGTACAG
N460A-F	AGAGCGCGCTGAAGCCTTTCGAGAGA
N460A-R	TTCAGCGCGCTCTTTCTGAACAGTCT
Y473A-F	AGATCGCGCAGGCCGGCAGCACACCG
Y473A-R	GCCTGCGCGATCTCGGTGCTGATGTC
S477A-F	CCGGCGCGACACCGTGTAATGGCGTG
S477A-R	GGTGTCGCGCCGGCCTGGTAGATCTC
F486A-F	AGGGCGCGAATTGCTACTTCCCTCTG
F486A-R	CAATTCGCGCCCTCCACGCCATTACA
N487A-F	GCTTCGCGTGCTACTTCCCTCTGCAG
N487A-R	TAGCACGCGAAGCCCTCCACGCCATT
Y489A-F	ATTGCGCGTTCCCTCTGCAGAGCTAC
Y489A-R	GGGAACGCGCAATTGAAGCCCTCCAC
Q493A-F	CTCTGGCGAGCTACGGCTTCCAGCCT
Q493A-R	TAGCTCGCCAGAGGGAAGTAGCAATT
Y505A-F	TGGGCGCGCAGCCTTACAGAGTGGTG
Y505A-R	GGCTGCGCGCCCACGCCATTGGTAGG
